# Can patients contribute to enhancing the safety and effectiveness of test‐result follow‐up? Qualitative outcomes from a health consumer workshop

**DOI:** 10.1111/hex.13150

**Published:** 2020-12-02

**Authors:** Judith Thomas, Maria R. Dahm, Julie Li, Andrew Georgiou

**Affiliations:** ^1^ Centre for Health Systems and Safety Research Australian Institute of Health Innovation Macquarie University North Ryde NSW Australia; ^2^ Institute for Communication in Health Care (ICH), College of Arts and Social Sciences Australian National University Canberra ACT Australia

**Keywords:** communication, consumer participation, diagnostic error, patient involvement, patient safety, test‐result follow‐up

## Abstract

**Background:**

Missed test‐results and failure to follow‐up test‐results are major patient safety concerns. Strategies to improve test‐results management have predominantly focused on clinician‐based interventions, with patients principally involved in studies of test‐result communication preferences, the impact of patient portals or experiences with reporting processes in primary care.

**Objective:**

To identify consumer perspectives and experiences of the challenges they have faced with test‐results management, through consumer participation in qualitative data analysis.

**Design and participants:**

Volunteers (n = 10) were recruited to participate in a health consumer reference group workshop on test‐results management. Prior to the workshop, consumers selected topics for discussion using a preference poll. During the workshop, consumers participated in qualitative data analysis of de‐identified excerpts of previously collected interview data discussing hospital test‐results management. Researchers (n = 5) guided consumers through the analytical process and discussion of themes. Discussions were audio‐recorded and transcribed for qualitative analysis.

**Results:**

Consumer‐selected topics for discussion were ‘Transitions of Care’ and ‘Access’. Consumer data analysis prompted broader discussion including lived experiences. Following the workshop, a second level of content analysis pinpointed issues with implications for patient safety highlighting that consumers were astutely aware of macrolevel ‘*Systems Factors’* relating to ‘*Emergency Departments’* and the health system, as well as microlevel ‘*Patient Factors’* (eg patient preferences and circumstances) which impact a patient's understanding during the ‘*Communication’* (clinician to patient/between clinicians) of test‐results ‘*Information’* (or lack thereof).

**Conclusions:**

Consumers identified the challenges patients experience with test‐results management, and our findings highlight areas for potential improvement in patient safety.

**Patient or public contribution:**

Ten health consumer volunteers actively participated in the test‐results management data analysis workshop conducted in this study. Two health consumers also volunteered to read and comment on the draft manuscript.

## 1 BACKGROUND

It is widely recognized that missed test‐results and failure to follow‐up test‐results are major patient safety concerns.^1^, ^2^, ^3^ Inadequate management of diagnostic test‐results can result in patient harm^2^, ^4^ or even death,^2^, ^5^ yet a study by Baylis et al^5^ of fifty general practice clinical negligence claims in the UK and Ireland (selected based on test‐results management system involvement) found that, in the majority of claims reviewed, the harm was avoidable.^5^ Avoidable harm represents an area where patient safety can be improved and there is an abundance of research into interventions aimed at improving safety through improving test‐results follow‐up.

A number of systematic reviews have been undertaken to examine evidence for effective interventions to improve the follow‐up of tests‐results including audit and communication strategies to reduce diagnostic errors^6^; interventions aimed at improving the follow‐up of test‐results pending at discharge (TPAD)^7^, ^8^; asynchronous laboratory results notifications^9^; and the management of test‐results using health information technology.^10^ The majority of articles examined in these systematic reviews have focussed on clinician‐centred strategies or interventions with only two reviews reporting studies of patient involvement in test‐results follow‐up, principally in relation to online access to test‐results via the Internet,^7^ patient portals^10^ or personal health records.^10^

As the recipients of diagnostic testing, patients have a vested interest in the effective management and communication of their test‐results. Furthermore, it is recognized that patients requesting or reviewing results may serve as a safety net for missed test‐results.^11^, ^12^, ^13^ Patient involvement in test‐results management has predominantly been researched from the perspective of patient preferences for test‐result communication^11^, ^14^, ^15^, ^16^ or patient experiences with accessing test‐results via web/patient portals,^17^, ^18^, ^19^, ^20^ in academic medical centres,^15^ outpatient,^14^, ^19^ primary care^11^, ^16^, ^19^ and hospital^18^‐based settings. Patient experiences with the total testing process^21^ or results management^22^ have been studied in primary care settings, identifying quality and safety themes^22^ and potential areas for practice process improvement.^21^ There is a notable deficit in literature reporting patient experiences of test‐results management in emergency department (ED) settings, despite the focus on interventions to improve TPAD in this context.^7^

Patient experience formed the premise of the current study which was undertaken as part of a larger project investigating test‐results communication, management and follow‐up.^23^ A health consumers (herein ‘*consumers’*) representative reference group was established to actively engage consumers to participate in qualitative data analysis during a workshop on test‐results management. The specific aims of the consumer reference group workshop (CRGW) were to

1. Identify key health consumer perspectives and experiences based on the challenges they have faced with test‐results management
2. Engage health consumers in qualitative health services research
3. Provide a forum where health consumers can participate in qualitative research to generate key themes related to test‐results management based on consumer selected priority topic areas

The rationale, practical and strategic approach to health consumer involvement in the CRGW has been reported elsewhere.^24^ The purpose of the current study was to report the outcomes and key findings from the CRGW focussing specifically on the first aim of the workshop, namely to ‘Identify key health consumer perspectives and experiences based on the challenges they have faced with test‐results management’, with the aim of identifying areas where patient safety could potentially be improved in consumer interaction with the management of test‐results.

## 2 METHODS

### 2.1 Context, study design and ethics

**FIGURE 1:**
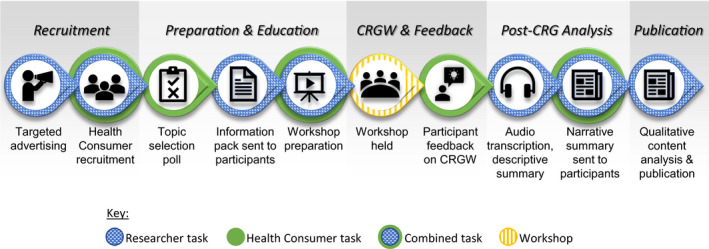
Workflow diagram of the study phases, tasks and roles. Consumers were recruited and then sent information packs prior to the workshop. A post‐workshop narrative summary was circulated to all participants

The CRGW was conducted in Sydney Australia, within the framework of a larger project investigating test‐results communication, management and follow‐up.^23^ The detailed strategies used in the planning and facilitation of this CRGW have been published elsewhere^24^; hence, only a summary is provided herein. A workflow diagram showing the study phases, tasks and roles performed by researchers and consumers is presented in Figure 1, with key tasks explained in the sub‐sections to follow.

Ethics approval was granted by the relevant Human Research Ethics Committee, and consumer volunteers provided written informed consent.

In the Australian context, public hospital emergency departments provide urgent care to patients presenting in person to the hospital or arriving via ambulance. No doctor's referral is required to attend a public hospital ED, and patients are triaged upon arrival. Most public hospital EDs are open 24 hours, 7 days a week, and treatment is provided at no cost to Australian Medicare card holders.^25^

### 2.2 Recruitment

Consumer representatives were recruited using targeted advertisements for volunteers, and the strategies underlying the targeted recruitment have been previously published^24^ in detail. Post‐recruitment, invitation letters including the workshop date, time, purpose, location, transport information and contact details for a member of the research team (the consumer ‘co‐ordinator’) were mailed out. To cover engagement costs, parking/cab vouchers were offered for travel assistance to attend the workshop (in line with Health Consumers NSW recommendations^26^).

Ten consumers (female n = 4) volunteered ranging between 40‐49 and 70‐79 (average = 61) years of age, including currently employed professionals and retirees. Self‐reported years of consumer experience ranged from 0 to 40+ (average 9.7 years). The CRGW was held in July 2018, and five researchers (female n = 4) also attended (including all authors).

### 2.3 Preparation‐ topic selection

Prior to the CRGW, recruited consumers were invited to select the topics for discussion at the workshop. Four topic areas related to the challenges associated with safe and effective test‐results management were offered for selection namely: (a) transitions of care; (b) patient‐facing care; (c) access; and (d) effect. A summary of the topic information provided to participants is presented in Appendix S1. Participants were asked to rank the four topics in order of relative importance and submit rankings via email or anonymously via a link to an online poll. Final topic selection was determined by assigning a priority score of 1‐4 (1 being highest priority) to each selection, summing the scores for each topic and then selecting the two topics with the lowest overall score.

All consumers completed the topic selection poll, and the results are presented in Appendix S2. Two respondents had 100% agreement in their rankings (Consumers 7 and 8). Based on consumers’ choices, the two topics with the lowest scores, Topic 1—transitions of care (score 2.5) and Topic 3—access (score 1.7), were selected for discussion at the workshop.

### 2.4 CRGW structure

The CRGW was a half‐day workshop held at Macquarie University with the first 30 minutes scheduled for introductions, housekeeping and a project overview presentation. For the analysis phase, participants were allocated to one of three tables (red table—‘RT’, blue table—‘BT’ and yellow table—‘YT) with a minimum of one researcher at each table.

Qualitative data analysis involved consumers reviewing interview excerpts specific to each topic area. The excerpts comprised previously collected de‐identified interview data from (a) emergency department patients and clinicians, (b) staff in pathology; and (c) staff in medical imaging departments discussing hospital test‐results management. These excerpts were provided to the consumers 10 days prior to the CRGW to allow time to read and reflect on the information.

The consumer co‐ordinator (Author 2) facilitated the workshop, and researchers at each table group guided consumers through the analytical process and discussion of potential themes. For each topic area, 10 minutes were allocated for consumer participants to individually re‐review data excerpts and annotate their initial thoughts/reactions. Approximately 35 minutes was allocated for each table group to engage in discussion regarding their thoughts and experiences, with one member of the table group acting as ‘scribe’ to note key comments and themes using ‘sticky notes’ or ‘butcher's paper’. Additional time was then allocated for each table to report a summary of their discussions to the broader group to facilitate an additional level of discussion. After a break in proceedings, the process was repeated for Topic 2. Following the two sessions, the facilitator provided an open ‘final thoughts’ opportunity for participants to provide any closing comments/opinions.

At the completion of the CRGW, each consumer was given a gift card as a gesture of appreciation for participating.^26^

### 2.5 Data collection

Multimodal data were collected across all phases of the project including consumer topic selection preferences; workshop audio recordings; artefacts including butcher's paper, sticky notes and whiteboard notes; and post‐workshop survey responses with written feedback. CRGW audio recordings were transcribed for analysis. Qualitative approaches used to maximize trustworthiness of the research findings included methodological triangulation^27^ using audio recordings, workshop artefacts and researcher participation to enhance data immersion. Investigator triangulation^28^, ^29^ involved five researchers attending the CRGW, of whom three performed the data analysis.

At the completion of the CRGW, consumers were asked to complete an anonymous feedback survey (Appendix S3). Results from the survey served as an additional data source for evaluating participant feedback on the engagement process.

### 2.6 Data analysis

The approach to data analysis was pragmatic and iterative. Data immersion involved three researchers (Authors 1‐3) reviewing the CRGW transcripts and artefacts to produce a narrative summary report for distribution to all participants. Upon completing this analysis, it was apparent that the workshop transcripts reflected both the participants’ involvement in, and the outcomes of, the analysis process with an abundance of microlevel themes. The discussions were also enriched with real‐life experiences/issues evoked by the analysis process. On this basis, it was determined that a second level of analysis was warranted to fully capture the richness of the CRGW proceedings. CRGW transcripts from the combined group discussions and ‘final thoughts’ session were subsequently analysed using qualitative content analysis as described by Grameheim^30^ to inductively derive meaning from the combined group discussion transcripts. Text was condensed and abstracted with key content presented on a purposely adapted Ishikawa diagram (Appendix S4). Content areas^30^ reflecting the manifest content of the data formed the ‘fishbones’, categories^30^ formed the ‘backbones’ and the ‘fish‐head’ was the resultant theme representing the latent content^30^ of the data. The three researchers repeatedly convened to review the textual/diagrammatic summaries until a consensus was reached on the final analysis.

Representative quotes have been de‐identified by referencing the table colour (‘BT’, ‘RT’ or ‘YT’) and topic being discussed (T1—topic 1, T2—topic 2). Individual consumer quotes from the ‘final thoughts’ transcript are prefixed with ‘CR’ (consumer representative) followed by consecutive numbers and gender, for example CR1‐male.

## 3 RESULTS

Five major themes were determined through qualitative content analysis including microlevel themes of (a) patient factors; (b) information; (c) communication; and macro level themes relating to the health system namely (d) ED factors and (e) systems factors, with a need for co‐ordination across all themes. Insights into test‐results management stemming from consumer participation in qualitative data analysis are detailed for each of the five themes. For each theme, the modified Ishikawa construct details the content informing each category and the categories underpinning each theme, with the exception of Theme 1—‘Patient Factors’, in which content was explained as representing a ‘spectrum’, and hence, the diagram is modified accordingly. The constructs and their relationship to each other are presented in their entirety in Appendix S5.

### 3.1 Patient factors

**FIGURE 2:**
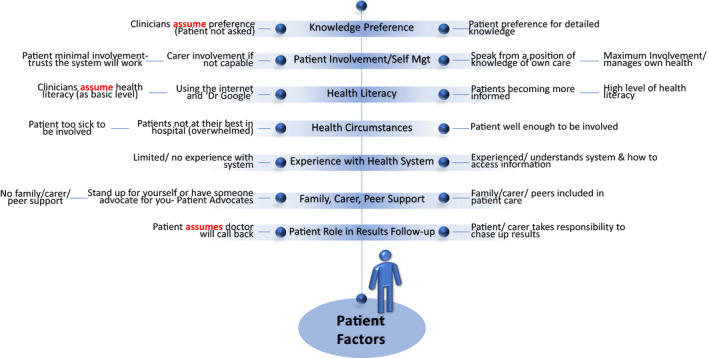
Patient factors determined from qualitative content analysis. Each factor represents a ‘spectrum’ of patients and an individual's position/preference for any factor may change with each episode of care depending on their circumstances

Consumers identified several patient factors that may impact on communication and a patient's understanding of information (Figure 2). Each factor represents a ‘spectrum’ of patients, and an individual's position/preference for any factor may change with each episode of care depending on their circumstances. These factors included knowledge preferences:

…it's important to ask patients what do you want to know, and not assuming that people either do want to know–some people want to know everything, some people don't want to know anything. (RT‐T1)

Health literacy was discussed at every table:

We also discussed health literacy…that patients are generally more informed…. (YT‐T1)

…it's not enough just to tell the patient or just to give people information, because it is going to depend on people's circumstances and their health literacy. (RT‐T1)

…about understanding, it depends on what, how is your information presented like what's your health literacy…. (BT‐T2)

It was also recognized that a patient's health circumstances can dominate their communication or information preferences:

…people aren't at their best when they're in hospital, so we maybe expect people to be able to communicate and take things on board when they're in hospital and by definition they're not at their best. (RT‐T1)

…there's a variation of patient expectations and ability from people who really want to know, really want to be involved, to people who don't want to know or actually, because they're sick and in hospital can't be involved. (RT‐T2)

Additional patient factors relating to family, carers, peer support and experience with the health system were discussed with one individual relating their experience:

…there are times when you're not yourself in ED…you don't know what's happening and you do need somebody there, especially family member…That knows your history…to be able to then fill in the dots where you are not necessarily with it…. (CR3‐male)

Some people don't have that. (CR2‐female)

A patient's involvement in their test‐results follow‐up, including whose responsibility it is to follow‐up test‐results, was discussed:

…it tends to be a patient's responsibility to chase up the results…. (BT‐T1)

…there are many assumptions about expecting a call back as well…I love the term ambiguity of responsibility, I mean, whose responsibility is it? Is it the ED physicians, is it your responsibility as a patient to chase that up or as a carer? (BT‐T1)

…we should have ownership of the tests, it's their intellectual property at the moment…when you've got an x‐ray you used to be able to get the sheet out of the x‐ray and you'd know what was wrong with you. Now it's all sent by email, it's nothing to do with you, you're completely cut out. (YT‐T2)

In summarizing discussions, patient involvement and self‐management were raised as key aspects:

…we sort of said that it's all about managing your own health, that takes into account all your different sort of levels of health literacy, the different levels of how involved you want to be…. (BT‐T1)

…patients being involved in their own care and having some knowledge about what's happening to them, so that they can speak from a position of knowledge. And ask questions. (RT‐T2)

### 3.2 Communication

**FIGURE 3:**
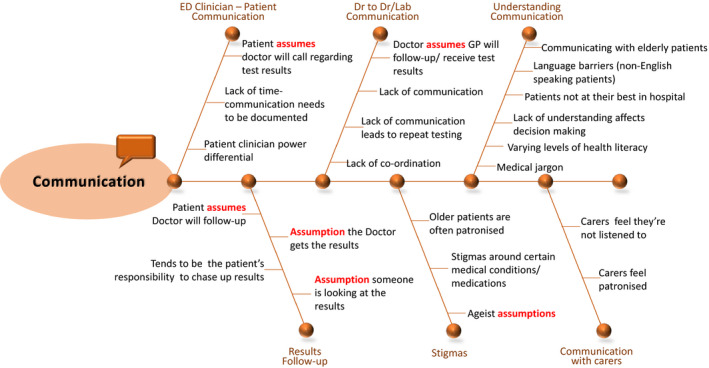
Communication theme, categories and content determined from qualitative content analysis

Communication (Figure 3) was discussed across different levels including clinician‐clinician, clinician‐patient, clinician‐carer and understanding communication. Content within the Communication theme was dominated by the term assume/assumption:

…there are many assumptions about expecting a call back…. (BT‐T1)

…the assumption that others will follow up or the assumption that we'll give you a call, whether the calls actually happen or not. (YT‐T1)

…doctors and pathology staff make the assumption that patient doesn't need to know, without consultation with the patients… (RT‐T2)

…the assumption that somebody's looking at the results…. (RT‐T1)

Negative aspects of communication were identified for both patients and their carers:

…older people in hospital are often patronised and there's quite a lot of ageist assumption involving people…. (RT‐T1)

…the issue of carers not being listened to and being treated in a patronising way…. (YT‐T1)

… one of the doctors [in the data excerpt said] that they'd really prefer that things go to the GP, but they're actually not realising that it actually puts the onus back on the patient to follow that up with the GP. (RT‐T2)

Perceived lack of communication between health‐care providers was seen to have negative impacts on patient safety:

…so having excess blood test results, excess imaging, when it might've been solved if there was more communication with all the teams to begin with. (BT‐T1)

…go to another hospital and another specialist, they have to re‐order the scan, so you're getting duplicate tests at quite high costs and probably worst for the person. …Yeah. Radiation. (YT‐T2)

In general, it was agreed that '*communication needs to be improved*' (BT‐T1) and '*what's needed is more consultation and a shared understanding of what's needed for this person at this time'*. (RT‐T2).

### 3.3 Information

**FIGURE 4:**
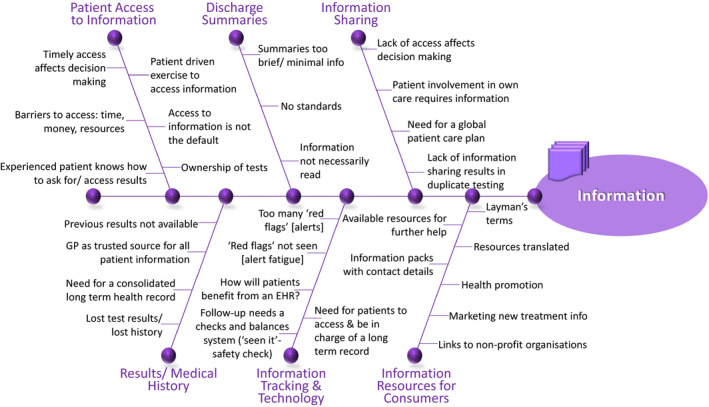
Information theme, categories and content determined from qualitative content analysis

Consumers concurred that access to information (Figure 4) is required for informed decision making:

…if you don't have access to your information in a timely manner, or you can't understand it or you can't take it to the place where it needs to be acted on appropriately, you can't really make the decisions appropriately or you can't follow why they made the decisions…. (BT‐T2)

The benefit of patient access to, and sharing of, information was also highlighted:

…what happens is that access to information, if you don't have it, it's one side the GP, the hospital, the specialist, so what they end up doing is doubling up, so you're wasting, so this is where patient access to information is actually a savings, because what you're doing is making the information available…. (RT‐T2)

However, it was apparent that access to information was not perceived as the default despite a patient desire for access and ownership of information. Requests for information were often associated with cost implications (eg time, money) if patients wanted ownership and involvement in the management of their health:

…access to information is usually not the default, so it's not by default you get access, you have to do something to get it, so it's usually a patient‐driven exercise and usually you have barriers and there's challenges to it, it costs time, it costs resources, it actually costs money in some cases…. (BT‐T2)

And basically it comes down to it's my body. If you want to touch it, that's fine but I have to give you permission to touch and if I want the information, I should […] have it. It's my body. (YT‐T2)

They further cautioned against clinicians making assumptions about patients’ health literacy and/or information needs. This was identified as particularly important given the increased vulnerability patients face in ED settings which often prompts family members or carers to adopt the challenging role of communication brokers to support patients in ED:

[I]t's important to ask patients "what do you want to know", and not assuming that […] people want to know everything, some people don't want to know anything. And it's not enough just to tell the patient or just to give people information, because it is going to depend on people's circumstances and their health literacy. (RT‐T1)

…so if you yourself are not capable you can assign it to a carer…. (BT‐T1)

…I actually had a family member who…did come in with me and knew everything because obviously I was just completely out of it. (CR3‐male)

The shortcomings of current systems were identified including discharge summaries and electronic information systems. Consumers also identified issues with health information and safety‐related consequences:

…there too many red flags and those red flags not being seen…. (YT‐T1)

…some people here had misdiagnosis or missed information in their treatment…. (BT‐T2)

…lost history and lost test results…. (BT‐T1)

A lack of targeted communication training and patient‐facing documentation and resources was identified. Being supported in understanding information or access to resources to assist with understanding was recommended:

…links to any sort of supporting information resources, not just to make sense of what your report says, but also to have resources for further help…. (BT‐T1)

…resources for consumer in terms of whether it may be acronyms to help with understanding or supporting documents or links to non‐profit organisations or where to seek more help, that should be automatically given to patients who've undergone treatment. (BT‐T2)

Participants challenged assumptions that discharge summaries contained quality information and that test‐results were transmitted seamlessly to their intended destination in a timely manner. In doing so, they highlight safety implications of missed or delayed information or a ‘dangerous’ reliance on a system:

…minimal information on discharge summaries. (BT‐T1)

…no standards [for discharge summaries]. (YT‐T1)

…a lot of doctors were assuming that this goes to the GP and we know that's not always borne out, the results don't always end up in the GP…. (RT‐T1)

Technology was seen as a potential benefit to information access:

…if you had a repository consolidating all your records that you are in charge of that you always have access, that you don't have to rely on your GP getting authorised to access that result…. (BT‐T1)

It should be noted that an electronic ‘My Health Record’ was emerging at the time of the CRGW with an opt‐out period for all Australians from July to October 2018.^31^

### 3.4 ED factors and systems factors

**FIGURE 5:**
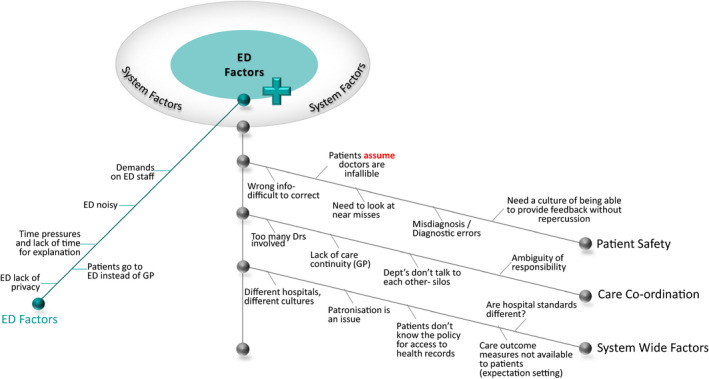
ED Factors and Systems Factors themes, categories and content determined from qualitative content analysis

Participants recognized that the ED is an environment where patients are either sick or injured and that this can impact their communication or understanding of information. Systems factors relating to the ED environment and the health system in general (Figure 5) were discussed including impact on information, communication and patient factors. Participants described how systemic time and resource pressure in ED impacted on the provision of test‐related explanation in ED with effects on the quality of care provided. Discussion relating to the ED setting identified:

…demands on staff and the pressures that people in ED are faced with. (YT‐T1)

…lack of time of explanation in the emergency department…. (BT‐T1)

No quiet space in most EDs and there's a lot of stigma as well…. (CR1‐male)

…duplication of results in ED…. (BT‐T1)

…there's only a curtain on either side and you can hear everything that's going on either side, there's no privacy for the patient and it's a huge issue…. (CR2‐female)

System‐wide factors, the need for care co‐ordination and patient safety were all recognized including:

…culture of being able to offer feedback because or to even ask questions…because the majority of people are apprehensive about clearing anything with staff in case there's repercussions…. (YT‐T2)

…policy about accessing health records…We think we should actually find out what that policy is. (RT‐T2)

…a lot of the data collection and the health sector is actually about process measures and efficiency. There's not a lot of measures about outcomes and certainly not a lot that's made available…. (YT‐T2)

…but it is a huge issue the number of mistakes that are made, we all assume that doctors are infallible…. (RT‐T2)

In recognition of the need for better communication and co‐ordination, one table proposed:

…different groups need to talk to each other and we thought standardised IT systems would help, as well as someone whose responsibility – someone in the hospital whose responsibility in the hospital is to pull it all together…. (RT‐T1)

### 3.5 Feedback survey

Feedback surveys were completed by 9/10 participants. All respondents indicated their expectations were met and that they would like to be involved in a similar event again. Respondents indicated they liked the open conversations, informative discussion, interesting approach and sense of equality and value. Suggestions for improvement included a bigger group, post‐forum networking opportunities and a longer workshop.

## 4 DISCUSSION

Through consumer participation in qualitative data analysis, the CRGW provided an in‐depth understanding of consumers’ experiences with test‐results management. The role of patients in the diagnostic process has been identified as an understudied area of research,^32^ and our study adds to existing literature in providing evidence from consumer experiences with a key component of the diagnostic process, namely test‐results management. Consumers highlighted a range of issues which have implications for patient safety and were astutely aware of both macrolevel ‘Systems Factors*’* relating to ‘ED Factors*’* and the health system in general, and individual microlevel ‘Patient Factors*’* which impact a patient's understanding in the ‘Communication*’* of test‐results ‘Information*’*.

Through insightful descriptions of patient‐related factors impacting test‐results management, an important issue was identified, namely the patient's role in results follow‐up. Consumers depicted patient involvement as a spectrum ranging from patients who assume a doctor will call, through to patients/carers who take responsibility to chase‐up results. Ambiguity regarding role delineation and assumptions regarding results follow‐up were identified, especially in the ED setting where patients may be discharged with pending test‐results. This raises a crucial patient safety question—who *is* responsible for ensuring results follow‐up? The need for policies and guidelines relating to responsibility for results follow‐up and notification is a long‐standing and pervasive issue in health care,^3^, ^33^ with an international survey of primary care practitioners finding ‘substantial variation’ in levels of follow‐up responsibility.^34^ Active patient engagement has been recognized as a positive strategy for reducing diagnostic error^32^, ^35^ and improving patient safety^32^ including test follow‐up.^13^ Consumers in the current study noting it '*tends to be the patient's responsibility to chase up the results'* and that hospital results being sent to their general practitioner (GP) put the *'onus back on the patient to follow that up'*; is suggestive that patients are actually acting as a safety net for results follow‐up. These statements are denotive of patients filling a potential safety ‘gap’ emerging from the ambiguity in responsibility for test‐results follow‐up or the ‘gaps’ between transitions of care^13^, ^36^ where our consumers were cognizant of prevailing ‘assumptions’ underlying the communication of test‐results.

Consumer discussion of communication in test‐results management depicted communication as a complex multifaceted theme. A major patient safety implication resulting from perceived shortcomings in communication between health‐care providers was repeat testing. The safety consequences of undergoing ‘'*excess blood test'*, '*excess imaging'*, '*duplicate tests'* and reordered scans included both cost implications and risks to patient health, for example '*radiation'*, which consumers believed could be avoided if communication was improved. This safety issue predominates across transitions of care^36^ where test‐results are not (or not able to be) communicated/shared between care providers. It is recognized in the literature that risks resulting from inadequate communication are not ‘widely appreciated’,^37^ yet our consumers were all too aware of the safety impacts on their own health. Consumers expressed a desire to have ownership of their test‐results, and greater involvement in managing their own health which, as recognized by McDonald et al,^38^ has the potential to act as another ‘safety net’ as patients can make their health information available to all clinicians involved in their care. Our findings support the need for greater recognition of the contributions patients can make^35^ to test‐results follow‐up^39^ and the ‘urgent need’ for improvement to results management.^33^

The inter‐relationship between ‘Communication’ and ‘Information’ was recognized by consumers who identified safety issues resulting from lack of access to information in a timely manner, including impacts on decision making and experiences of ‘misdiagnosis’ or ‘missed information’ in their treatment. Electronic access to test‐results/patient health records was not widely available in Australia at the time of our study and hence consumers perceived such technology as beneficial to information access. Consumers also recognized the need for information resources to support understanding health information (eg defining acronyms) to both ‘make sense’ of test reports and as reliable resources for further help. This finding is consistent with research into the impact of patient portals which has identified the need to ensure patients understand their test‐results^19^ through, for example, the addition of provider interpretation^19^/comments,^17^ or portal links to additional resources.^17^, ^19^

Consumer involvement in qualitative data analysis has been identified as an understudied area of research.^40^ To the best of our knowledge, our study is the first to engage consumers in test‐results management qualitative data analysis. The CRGW outcomes were an intertwining of issues, discussion points and personal experiences. Collating the consumers' analysis into higher level themes (from microlevel subthemes) was not achieved within the timeframe of the CRGW and represents a lesson learned for future workshops. Upon reflection, it may have been more constructive to reduce the analysis to a single topic with greater focus on achieving thematic outcomes. However, the microlevel of thematic analysis painted a vivid picture of consumers’ experiences and provided invaluable insight into the role that patients may be undertaking in acting as a safety net for missed test‐results. From a methodological and analytical standpoint, the CRWG also presented innovative opportunities to include interpretative lenses outside traditional health service research and academia; not just recording the patient voice on the frontline but ensuring consumers’ voices is represented in data analysis as well.^24^, ^41^, ^42^ The collaborative analysis and identification of additional threats to quality and safety highlight the complexity of the diverse realities faced by patients. Our experiences highlight the immense potential that can be achieved through affording consumers with opportunities for active partnerships in health service research.

### 4.1 Limitations

A major limitation of this study was recruitment of volunteers and as such the results are reflective of those who agreed to participate, who were consumers with experience and/or an interest in this field. As such, it is recognized that our volunteers may not be 'truly representative of the targeted population',^43^ ^pg. 7^ that is all health‐care consumers, and this may have impacted the study findings. It is acknowledged that, due to the small number of participants, minority representation was not achieved in this study^44^ and our study lacked representative sampling from the <35‐year age group.

## CONFLICT OF INTEREST

The authors have no conflicts of interest to declare.

## AUTHOR CONTRIBUTIONS

All authors made valuable contributions to the study. AG and MD designed and developed the study. MD served as the consumer co‐ordinator throughout the study. JL, JT, AG and MD were researchers present at the CRGW. JT, MD and JL performed the data analysis, and JT drafted the manuscript. AG, JL, JT and MD critically reviewed the manuscript for important intellectual content and approved the final manuscript.

## Supporting information

Appendix S1

Appendix S2

Appendix S3

Appendix S4

Appendix S5

## Data Availability

The author elects to not share data: Research data are not shared based on the requirements of the ethics body.
