# Supplementary material for: Can patients contribute to enhancing the safety and effectiveness of test‐result follow‐up? Qualitative outcomes from a health consumer workshop
Source: Health Expect. 2020 Dec 2;24(2):222–33. doi: 10.1111/hex.13150 (PMC8077113; doi:10.1111/hex.13150)
Supplement: Supplementary file 1 — Appendix S1 [file HEX-24-222-s005.docx]

Appendix S1: Four Topics for Prioritisation by CRGW Participants

| Topic | Description |
| --- | --- |
| 1. Transitions of care   *“I am aware of the next steps in my care”* | Communication is often fragmented between care environments within hospitals (e.g., between Pathology and the emergency department) and across care settings (e.g., from hospital to GP). Test results are not efficiently communicated to doctors, patients and across the health spectrum. Inefficient communication of (outstanding) test results is a risk to patient safety and the continuity of care. |
| 1. Patient-facing care   *“My care preferences are being taken into consideration”* | Every patient is different, and their care needs are varied. Information should be truly tailored to each patient considering personal preferences and characteristics. |
| 1. Access   *“Do I have the information I need to understand the decisions being made?”* | Patient access to test result (if desired) offers opportunities for patient empowerment, greater ownership of care. |
| 1. Effect   *“What impact does test results management have on my well-being?”* | The way test results are managed can have an impact on the way in which patients experience the care they receive. |
