# Supplementary material for: Can patients contribute to enhancing the safety and effectiveness of test‐result follow‐up? Qualitative outcomes from a health consumer workshop
Source: Health Expect. 2020 Dec 2;24(2):222–33. doi: 10.1111/hex.13150 (PMC8077113; doi:10.1111/hex.13150)
Supplement: Supplementary file 2 — Appendix S2 [file HEX-24-222-s004.docx]

Appendix S2: Participant Topic Prioritisation Ranking Scores

| Respondent | Topic 1-  Transitions of care | Topic 2-  Patient-facing care | Topic 3-  Access | Topic 4-  Effect |
| --- | --- | --- | --- | --- |
| Consumer 1 | 1 | 3 | 2 | 4 |
| Consumer 2 | 3 | 4 | 1 | 2 |
| Consumer 3 | 4 | 2 | 3 | 1 |
| Consumer 4 | 1 | 2 | 3 | 4 |
| Consumer 5 | 3 | 1 | 2 | 4 |
| Consumer 6 | 3 | 4 | 2 | 1 |
| Consumer 7 | 2 | 3 | 1 | 4 |
| Consumer 8 | 2 | 3 | 1 | 4 |
| Consumer 9 | 4 | 2 | 1 | 3 |
| Consumer 10 | 2 | 4 | 1 | 3 |
| Sum of Scores: | **2.5** | **2.8** | **1.7** | **3** |
| Priority Ranking: | **2nd** | **3rd** | **1st** | **4th** |

(1= Highest, 4= Lowest)
