# Supplementary material for: Can patients contribute to enhancing the safety and effectiveness of test‐result follow‐up? Qualitative outcomes from a health consumer workshop
Source: Health Expect. 2020 Dec 2;24(2):222–33. doi: 10.1111/hex.13150 (PMC8077113; doi:10.1111/hex.13150)
Supplement: Supplementary file 3 — Appendix S3 [file HEX-24-222-s003.docx]

Appendix S3- Post-CRGW Survey Questions

1. What were your expectations for the meeting today? Were your expectations met?
2. Have you ever attended an event with a research focus like this?
3. What did you think about the quality of the information material sent ahead of the meeting?
4. What did you like about today?
5. What could have been improved?
6. Would you get involved in a similar event again? Why?
7. Anything else you would like to share?
8. Age
9. Gender
10. What is your profession?
11. Consumer representative experience (months/years)
