# Supplementary material for: Can patients contribute to enhancing the safety and effectiveness of test‐result follow‐up? Qualitative outcomes from a health consumer workshop
Source: Health Expect. 2020 Dec 2;24(2):222–33. doi: 10.1111/hex.13150 (PMC8077113; doi:10.1111/hex.13150)
Supplement: Supplementary file 5 — Appendix S5 [file HEX-24-222-s001.docx]

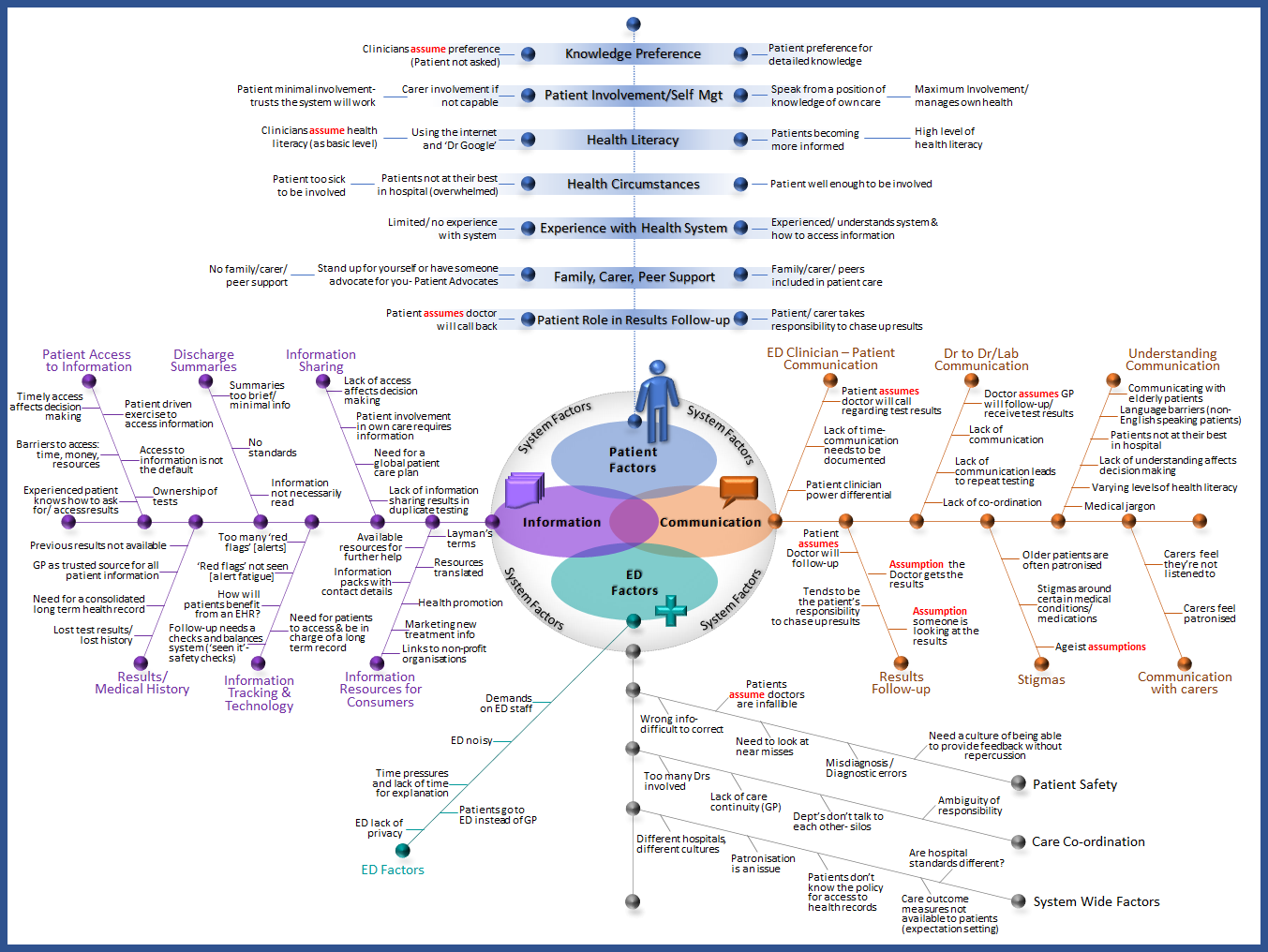
Appendix S5- Summary Diagram of Qualitative Content Analysis Using Modified Ishikawa Constructs
